# The course of hepatitis E virus infection in pigs after contact-infection and intravenous inoculation

**DOI:** 10.1186/1746-6148-5-7

**Published:** 2009-02-04

**Authors:** Martijn Bouwknegt, Saskia A Rutjes, Chantal BEM Reusken, Norbert Stockhofe-Zurwieden, Klaas Frankena, Mart CM de Jong, Ana Maria de Roda Husman, Wim HM van der Poel

**Affiliations:** 1 Laboratory for Zoonoses and Environmental Microbiology, Centre for Infectious Disease Control Netherlands, National Institute for Public Health and the Environment, PO Box 1 (pb. 63), NL-3720BA, Bilthoven, The Netherlands; 2 Quantitative Veterinary Epidemiology Group, Wageningen Institute of Animal Sciences, Wageningen, The Netherlands; 3 Central Veterinary Institute, Wageningen University Research, Lelystad, The Netherlands

## Abstract

**Background:**

Worldwide, hepatitis E virus (HEV) genotype 3 is observed in pigs and transmission to humans is implied. To be able to estimate public health risks from *e.g*. contact with pigs or consumption of pork products, the transmission routes and dynamics of infection should be identified. Hence, the course of HEV-infection in naturally infected pigs should be studied.

**Results:**

To resemble natural transmission, 24 HEV-susceptible pigs were infected either by one-to-one exposure to intravenously inoculated pigs (C1-pigs; n = 10), by one-to-one exposure to contact-infected pigs (C2-pigs: n = 7; C3-pigs: n = 5) or due to an unknown non-intravenous infection route (one C2-pig and one C3-pig). The course of HEV-infection for contact-infected pigs was characterized by: faecal HEV RNA excretion that started at day 7 (95% confidence interval: 5–10) postexposure and lasted 23 (19–28) days; viremia that started after 13 (8–17) days of faecal HEV RNA excretion and lasted 11 (8–13) days; antibody development that was detected after 13 (10–16) days of faecal HEV RNA excretion. The time until onset of faecal HEV RNA excretion and onset of viremia was significantly shorter for *iv*-pigs compared to contact-infected pigs, whereas the duration of faecal HEV RNA excretion was significantly longer. At 28 days postinfection HEV RNA was detected less frequently in organs of contact-infected pigs compared to *iv*-pigs. For contact-infected pigs, HEV RNA was detected in 20 of 39 muscle samples that were proxies for pork at retail and in 4 of 7 urine samples.

**Conclusion:**

The course of infection differed between infection routes, suggesting that contact-infection could be a better model for natural transmission than *iv *inoculation. Urine and meat were identified as possible HEV-sources for pig-to-pig and pig-to-human HEV transmission.

## Background

Hepatitis E virus (HEV) is a positive sense, non-enveloped single-stranded RNA virus with a genome of 7.2 kb and can be grouped into at least four genotypes [1]. Hepatitis E virus was considered to be restricted to developing countries, but it is now considered an emerging pathogen in developed countries [e.g. [2]]. The epidemiology of HEV, however, differs between developed and developing countries [1]. In developing countries all four genotypes of HEV are found in locally acquired hepatitis E cases, whereas in developed countries locally acquired HEV cases are caused by genotypes 3 and 4 [3]. HEV infections with genotypes 1 and 2 are implicated in both epidemic and sporadic cases of HEV infection, whereas genotypes 3 and 4 have been only implicated in sporadic cases so far. Sources for these sporadic cases in industrialized countries are uncertain. The absence of person-to-person transmission of HEV genotype 3 among 18 household members of acute hepatitis E patients in the Netherlands [4] suggests that human HEV infections acquired in the Netherlands are of environmental origin rather than person-to-person transmission. Worldwide, HEV has been reported in environmental sources, including surface water [5], animal species including domestic pigs and wild boar [6], sewage of animal origin [7], and foods of animal origin [8-12]. Zoonotic foodborne HEV transmission via wild deer meat has been proven [13], but not from other environmental sources. An increased anti-HEV seroprevalence in people working professionally with pigs [14,15] and presence of infectious HEV in commercial porcine livers at retail [11] however, suggests that swine may be a source of human exposure to HEV. Based on the phylogenetic similarity between HEV-sequences from human and swine, interspecies transmission was suggested [16,17].

In the Netherlands, about 7.5 × 10^6 ^fattening pigs are raised annually [18]. HEV RNA was observed in faeces from >50% of Dutch fattening pig farms [19] and HEV has a high transmission potential among domestic swine [20]. Therefore, domestic swine may be an important reservoir for human HEV infections, but to which extent is currently unknown. To be able to estimate the public health risk using field data on the occurrence of HEV in pigs, the natural course of HEV infection in pigs needs to be known. Several studies have presented experimental data for intravenously (*iv*) inoculated pigs, showing onset of faecal HEV RNA excretion at one to two weeks postinoculation and onset of viremia at two to three weeks postinoculation [21-25]. Faecal HEV RNA excretion may last up to 7 weeks, whereas viremia is detected generally for one to three weeks [22,24]. Faecal HEV RNA excretion is observed in all pigs after *iv *inoculation, but viremia and antibody development are not observed in all *iv *inoculated pigs. Antibodies to HEV infection are detected between two and eight weeks postinoculation [22,23,26]. Increased liver enzyme levels in serum are generally not observed in *iv *inoculated pigs [21-23,25].

However, whether *iv *inoculated pigs display a course of HEV infection that resembles that of naturally infected pigs (presumably via the faecal-oral route [1]) is currently unknown. Direct oral inoculation of HEV in pigs has been unsuccessful in all but one pig [24]. This pig received a dose of at least 10^8 ^HEV genome equivalents (one genome equivalent was defined as the number of HEV genomes present in the highest serial dilution positive by RT-PCR), whereas two other pigs that received this dose remained uninfected. Contact-exposure of a susceptible pig with an infectious pig appears to lead to HEV infection more easily than direct oral inoculation [22-24]. Thus, the use of contact-infected pigs may be a good alternative to study the course of HEV infection for naturally infected pigs. Contact-infected pigs are likely to reflect the natural course of HEV-infection more accurately than *iv *inoculated pigs.

The aim of the current study was to describe the course of HEV infection in contact-infected pigs by estimating the time until and the duration of faecal HEV RNA excretion and viremia and the time until antibody development. Localization of HEV in the pig and liver enzyme levels in serum were also assessed. As *iv *inoculated pigs were used to generate first-, second- and third-generation contact-infected pigs [20], the data for contact-infected pigs were compared to those for *iv *inoculated pigs to identify possible differences in the course of infection due to infection route.

## Results

### Statistics describing the course of HEV infection

The time until and the duration of faecal HEV RNA excretion and viremia, and the time until antibody development were estimated for contact-infected and *iv*-pigs (Table 1). No statistical differences were observed between C1-pigs and C2/3-pigs (data not shown), and therefore data for all contact-infected pigs were pooled. Statistical differences between the two blocks were observed for the five parameter estimates. Statistical differences were observed between contact-infected pigs and *iv*-pigs for two parameters in both blocks, whereas one parameter differed significantly between contact-infected pigs and *iv*-pigs in one block only (Table 1). To conclude whether an overall difference between contact-infected pigs and *iv*-pigs existed, joint *p*-values were calculated. Overall differences were observed for time until onset of faecal HEV RNA excretion and viremia, and duration of faecal HEV RNA excretion.

**Table 1:** Differences in five parameters describing the course of HEV-infection in contact-infected and *iv *inoculated pigs

			*P*-values		
			
Days...	Contact-infected	Intravenously inoculated	Block 1	Block 2	Joined*
until faecal HEV RNA excretion	7.2 (4.8 – 9.6)	3.2 (2.0 – 4.3)	0.013	0.020	0.002
with faecal HEV RNA excretion	23.3 (18.7 – 27.9)	39.9 (27.7 – 52.1)	0.021	0.275	0.036
until viremia^†^	12.6 (8.3 – 17.0)	3.8 (2.2 – 5.4)	0.052	0.048	0.017
with viremia	10.5 (8.1 – 13.0)	26.2 (16.6 – 35.8)	0.089	0.345	0.137
until antibody development^†^	13.0 (10.3 – 15.6)	12.5 (10.4 – 14.6)	0.067	0.300	0.097

* Fisher's joint *p*-value

^† ^since first faecal HEV RNA excretion

Statistical differences were absent among C1-, C2- and C3-contact infected pigs for these five parameters, for which the data for contact-infected pigs were pooled.

**Figure 1:**
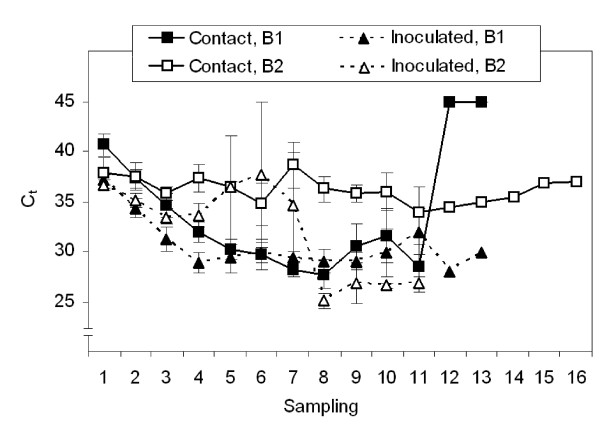
**The pattern of faecal HEV RNA excretion in time**. The pattern is represented by the threshold cycle (C_t_) of the real-time RT-PCR per block (B1: Block 1, B2: Block 2) for contact-infected and *iv *inoculated pigs. The C_t _values were used as relative marker for the amount of HEV in samples, under the assumption that efficiencies of the assay for all faecal samples are comparable. The first HEV-positive faecal sample is taken as starting point of the pattern (*i.e*. sampling 1) and three samplings represent 7 days. C_t _values were averaged for contact-infected or *iv *inoculated pigs per sampling. Error bars indicate the standard error of the mean; absence of error bars means that only one value was available for that sampling. Note that a lower C_t _usually indicates a higher HEV-concentration and C_t _>40 were set at 45, because 45 cycles were completed in the RT-PCR assay.

**Figure 2:**
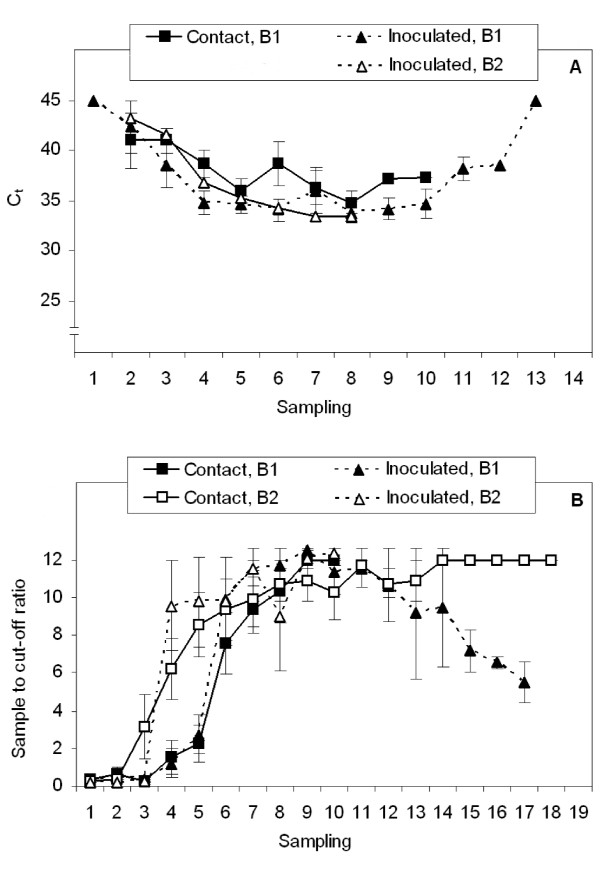
**The pattern of HEV viremia (A) and anti-HEV antibody (total Ig) development for contact-infected pigs and *iv *inoculated pigs (B)**. The first HEV-positive faecal sample is taken as starting point of the patterns (*i.e*. sampling 1) and two samplings represent 7 days. C_t _and sample to cut-off ratios were averaged for contact-infected or *iv *inoculated pigs per sampling. Note that a lower C_t _usually indicates a higher HEV-concentration and C_t _>40 were set at 45. Error bars indicate the standard error of the mean; absence of error bars indicates that only one value was available for that time-point. As only four contact-infected pigs in Block 2 showed viremia with scattered times of onset, no graph could be produced for these pigs.

From the first faecal HEV RNA excretion onwards, the course of the HEV RNA-titre in faeces and serum, and the development of detectable antibodies were similar for *iv*-pigs and contact-infected pigs (Fig. 1 and 2).

### AST and ALT levels in serum

No instant elevations in serum ALT- or AST-levels were observed for the seven contact-infected pigs and five *iv-*pigs when the values obtained were related to the baseline values for fattening pigs (data not shown). However, the presence of an increasing trend was assessed per pig by ordinary linear regression, showing a statistically significant (*p *< 0.05) increase in serum ALT-levels during the period of faecal HEV RNA excretion for one contact-infected pig necropsied 55 days after first faecal HEV RNA excretion and for three *iv*-pigs (nos. 6, 7 and 9 in Tables 2 and 3). The differences in minimum and maximum values for these pigs were 14, 42, 38 and 29 U l^-1^. The other 8 pigs did not show an increasing trend for ALT-levels and none of the pigs for AST-levels.

**Table 2:** HEV RNA in organs, excreta and bile from second-generation contact-infected pigs and *iv *inoculated pigs at 28 days post infection.

	Contact infected						Inoculated					
		
Pig	1	2	3	4	5	Total	Total	6	7	8	9	10
Faeces	+	-	-	-	+	2/5	5/5	+	+	+	+	+
Urine	+	-	-	+	+	3/5	0/5	-	-	-	-	-
Serum	-	-	-	-	+	1/5	4/5	+	+	-	+	+
Bile	+	-	-	-	+	2/5	5/5	+	+	+	+	+
Liver	+	-	-	-	+	2/5	5/5	+	+	+	+	+
Mesenterial LN	+	-	-	-	+	2/5	5/5	+	+	+	+	+
Bronchial LN	+	-	-	-	+	2/5	5/5	+	+	+	+	+
Hepatic LN	+	-	-	+	+	3/5	5/5	+	+	+	+	+
Pancreas	-	-	-	-	-	0/5	0/5	-	-	-	-	-
Spleen	+	-	-	+	+	3/5	5/5	+	+	+	+	+
Kidney	-	-	-	-	+	1/5	4/5	+	+	-	+	+
Ileum	-	-	-	-	.	1/4	4/5	+	+	-	+	+
Jejunum	-	-	-	-	+	1/5	4/4	+	+	.	+	+
Colon	+	-	-	-	+	2/5	4/5	+	+	-	+	+
Tonsil	-	-	-	-	-	0/5	3/5	+	-	-	+	+

'+' indicates the presence of HEV RNA; '-' indicates the failure to detect HEV RNA; '.' indicates that samples were not examined

**Table 3:** HEV RNA in samples of muscle, liver and serum at various days since first faecal-HEV excretion in contact-infected pigs (C1, C2 and C3) and intravenously inoculated pigs (*iv*).

			Type of muscle				
					
Day*	Type	Pig ID	Longissimus	Biceps femoris	Iliopsoas	Liver	Serum
13	C3	21	+	+	-	+	.
13	C3	22	+	+	+	+	+
15	C3	23	+	+	+	+	+
18	C3	24	-	-	-	-	-
19	C2	19	+	+	+	+	.
23	C1	15	-	-	+	+	+
24	C1	16	+	+	+	+	.
25	*iv*	6	-	+	+	+	+
25	*iv*	7	+	+	+	+	+
25	*iv*	8	-	+	-	+	-
25	*iv*	10	+	+	+	+	+
27	C2	3	-	-	-	+	-
27	C2	5	+	+	+	+	+
27	*iv*	9	+	+	+	+	+
29	C1	17	-	-	-	-	.
30	C2	4	-	-	-	-	-
31	C1	18	-	+	-	-	.
32	C2	20	-	-	+	+	+
53	*iv*	14	-	-	-	-	-
55	*iv*	13	-	-	-	-	-

'+' indicates the presence of HEV RNA; '-' indicates the failure to detect HEV RNA; '.' indicates that samples were not examined

* since first faecal HEV RNA excretion

### HEV RNA in tissues, urine and bile

Five C2-pigs and five *iv*-pigs were necropsied at 28 days post infection to collect tissue samples, urine and bile (Table 2). HEV RNA was found less often in samples of C2-pigs (24 of 74) than in samples of *iv*-pigs (58 of 74). Remarkably however, urine of three C2-pigs tested HEV RNA-positive (with C_t _values of 37, 38 and 31 for pigs no. 1, 4 and 5, respectively), as opposed to none of the *iv*-pigs. Sequences of the RT-PCR fragments were obtained for the three samples by cloning and sequencing, and were homologous to the inoculated HEV strain.

At 56 dpi, HEV RNA was detected in urine, the bronchial and hepatic lymph nodes and the spleen of both *iv*-pigs. Furthermore, HEV RNA was present in the faeces of one of the two pigs, the other showed HEV RNA in the ileum, jejunum and the mesenteric lymph node. The C1-pig necropsied at 55 days after first faecal HEV RNA excretion showed HEV RNA in the kidney and the C1-pig necropsied at 65 days showed HEV RNA only in urine. Interestingly, both urine specimens of the *iv*-pigs and one urine specimen of the C1-pig contained HEV RNA at necropsy. The urine specimen of the *iv*-pigs both showed a C_t _>40, whereas the specimen of the C1-pig showed a C_t _of 32.5. This C1-pig had stopped excretion of HEV in faeces about 4 weeks earlier. Clones with correct inserts were obtained for RT-PCR fragments detected in urine of the C1-pig. Subsequent sequencing showed homology to the HEV sequence of the inoculated strain.

### HEV RNA in muscle samples

HEV RNA was detected in 12 of 20 biceps femoris muscle samples, in 11 of 20 iliopsoas muscle samples and in 9 of 20 longissimus muscle samples at various time-points after infection (Table 3). Muscle samples were found positive in contact-infected pigs up until 32 days after first faecal HEV RNA excretion. Out of 14 liver-positive pigs, 13 tested positive in at least one muscle sample (and eight in all three muscle samples), while five out of six liver-negative animals tested negative in all muscle samples. In addition, HEV RNA was detected in examined serum samples in all but one pig (no. 8) that showed HEV RNA-positive muscle samples.

### Histopathology

No gross pathological changes were observed in any of the examined pigs. By histopathology, moderate, multifocal, lymphohistiocytic hepatitis was observed in livers from two of five C2-pigs (nos. 4 and 5 in Table 2) and three of five *iv*-pigs (nos. 6, 9 and 10) after necropsy at 28 days postinfection. In addition, slight subepithelial lymphohistiocytic cell infiltrations were observed in the gall bladder of one C2-pig and one *iv*-pig necropsied at 28 days post infection, and of one C1-pig necropsied at 55 days after first faecal HEV RNA excretion. In the ileum a mild or moderate hyperplasia of Peyer's patches was observed in four of four examined C2-pigs, four of four examined *iv*-pigs and three of four pigs necropsied at the end of the study. A mild or moderate hyperplasia in lymph nodes was observed in one C2-pig (no. 5), two *iv*-pigs (nos. 6 and 7) and the C1-pig necropsied 65 days after first faecal HEV RNA excretion. No lesions were observed in the spleen and pancreas.

## Discussion

In the current study, HEV infection in pigs due to contact-exposure to HEV-infectious pigs was studied as model for natural transmission to describe the natural course of HEV infection in pigs. The use of intravenous HEV inoculation as initiator of the transmission process enabled collaterally the identification of differences in the course of infection due to infection route. Furthermore, HEV RNA was detected in several porcine tissues, muscles and excreta.

We present evidence of HEV RNA in urine in the current study, with 6 of 14 urine samples containing HEV RNA compared to 8 of 14 faecal samples collected at the same time. A single observation of HEV RNA in urine of a pig was reported before by Banks *et al*. [27]. Because urine samples in the current study were collected directly from the bladder using a sterile syringe with needle, contamination by HEV from other sources is unlikely. One HEV RNA-positive urine sample was obtained from a contact-infected pig at 65 days after onset of faecal HEV RNA excretion, showing a comparable C_t _to that of faecal samples collected during the acute phase of infection. This finding may suggest that HEV-excretion via urine lasts longer than faecal HEV RNA excretion, and/or that urinal HEV-excretion occurs at a later stage of infection. This issue should be resolved by monitoring longitudinally the presence of HEV in urine of contact-infected pigs.

The presence of HEV RNA in urine suggests that HEV may be transmitted via urine. Until now, faecal-oral transmission of HEV is considered the main transmission route among pigs. The possibility of other transmission routes has been discussed [21,22,26], but efforts to experimentally transmit HEV between pigs via tonsil and nasal secretions, and HEV-contaminated needles have been unsuccessful [24]. The physical condition of urine yields easier distribution of HEV throughout a pen or stable than with faeces. In addition, the amount of urine excreted per pig per day is 5-fold the amount of excreted faeces [28]. Transmission of HEV via urine might occur orally, or aerogenically via droplet aspiration to the respiratory tract. For urine to be a transmission route, however, the HEV RNA should originate from infectious HEV particles, which is currently unknown. The detected HEV RNA may represent HEV that is bound to antibodies for disposal; anti-HEV antibodies have been observed in urine samples of human hepatitis E patients [29]. Whether or not urine contains infectious HEV could be examined by performing cell infection experiments [30,31] or experimental inoculation of susceptible pigs with a HEV-positive urine sample.

HEV RNA was detected in samples from organs and muscle. These samples may contain HEV RNA either extrinsically (on the surface) due to cross-contamination during necropsy or intrinsically (within the tissue). During necropsy, the organs containing excreta (faeces and bile) that can harbour high loads of HEV were sampled last to reduce the potential for extrinsic contamination. Although the contribution of these excreta cannot be excluded entirely, the most likely source for extrinsic contamination is blood. This reasoning holds especially for the muscle samples, as these were taken outside of the abdominal cavity before any other organ sample was collected. Indeed, eight of nine examined pigs with HEV-contaminated muscle samples were viremic, suggesting a potential role of blood in contamination. Whether this role involves intrinsic and/or extrinsic contamination remains to be examined, for instance by immunohistochemistry [32].

More than 50% of the muscle samples examined in the current study contained HEV RNA. Previously, HEV RNA was detected in skeletal muscle in pigs that were inoculated *iv *with HEV of genotype 3 obtained from a human patient, but not with HEV from swine [33]. The muscle samples examined in the current study were proxies for commercial pork meat (pork steak, tenderloin or pork chop). Positive samples were found until four weeks after onset of faecal HEV RNA excretion, which suggests that HEV RNA-contaminated meat could only enter stores when new HEV-infections occur later in the fattening period. For Japanese pig farms it was estimated that about 5% of the pigs were infected after five months of age [34]. In a Dutch slaughterhouse, 14% out of 80 sampled pigs excreted HEV faecally, suggesting these pigs were in the acute phase of infection (Rutjes *et al*., unpublished data). From a public health perspective, it should be considered whether contaminated pork meat at retail indeed contains infectious HEV and what fraction remains infectious until consumption. It is advisable, however, to cook pork properly prior to consumption.

In the current study, the course of HEV infection in pigs was described for contact infected pigs. Simultaneously, these data were collected from *iv *inoculated pigs, enabling comparison between the two. The duration of faecal HEV RNA excretion was exceeded by about 16 days for *iv-*pigs compared to contact-infected pigs. In contrast, the delay in onset of faecal HEV RNA excretion was reduced by about 4 days for *iv*-pigs compared to contact-infected pigs. This reduction may, however, be partially explained by the required exposure-time for infection, which is absent in the estimate for *iv*-pigs. From the current transmission study, the rate of transmission per day was estimated at 0.66 (95% confidence interval: 0.32 – 1.35) [20]. The reciprocal of this figure gives the average exposure-time required for infection, which equals 1.5 (0.7 – 3.1) days. With the delay being 4.1 days (95% CI: 0.6 – 7.6), the exposure time explains only partially the delay. The remainder may be explained by the difference in route of infection. Other differences possibly attributable to route of infection were time until viremia and the higher number of HEV RNA positive organs in *iv*-pigs. The observed differences suggest a more severe infection after *iv *inoculated pigs compared to contact-infected pigs. Whether these differences are important to consider in planning future experiments will depend on the aim. For instance, when using swine as a bioassay to detect infectious HEV [26] these differences will not be important, whereas in risk assessment studies the risks needs to be estimated as accurately as possible and the differences are important.

The HEV titres in serum and faeces were similar between *iv*-pigs and contact-infected pigs in the current study once faecal HEV RNA excretion had started. Meng *et al*. [23] described that HEV titres in faeces increased about 2 log_10 _(representing 6.6 C_t _units under ideal real time RT-PCR conditions) when the inoculated HEV-dose increased 1,000-fold. This relationship would suggest that the HEV-doses for contact-infected pigs and *iv*-pigs in the current study were comparable and that contact-infected pigs ingested sufficient HEV to result in about 10^4 ^HEV RNA particles entering the bloodstream. Variation in ingested HEV-doses, however, seems likely for contact-infected pigs, because their ingestion of HEV was uncontrolled. If the doses differed, then similar HEV titres for contact-infected and *iv*-pigs suggest a plateau level of HEV released by infected cells. It has been hypothesized that HEV-release from infected cells may be caused by the immunological response rather than the cytopathic effect of HEV on hepatocytes [35]. In this perspective, the apparent plateau might reflect the maximal effect of the immune system. The current data, however, leave this issue unresolved.

Antibody development was detected in the current study at the earliest at about two days after first faecal HEV RNA excretion. The average time until antibody development was two weeks after first faecal HEV RNA excretion (contact-infected pigs) or inoculation (*iv*-pigs). Reported times until IgG development for *iv *inoculated pigs were two weeks after inoculation at the earliest [21,23], but more frequently reported are times between three and eight weeks postinoculation [22,26]. The difference between previous studies and the current study may be caused by the principle of the ELISAs used. In the current study, the double antigen sandwich ELISA detects IgM and IgA in addition to IgG. The IgM antibody contains 10 antigen binding sites as opposed to two for IgG, for which the likelihood to bind at least one immobilized antigen and one conjugated antigen is higher for IgM than for IgG, as discussed by Rutjes *et al*. (unpublished data). As the onset of IgM generally precedes the onset of IgG after infection, total antibody development is likely detected earlier than IgG development only. This advantage could be used in *e.g*. seroprevalence screening to minimize misclassification of the subject due to the delay in antibody development.

Histopathological lesions were observed in the liver, gall bladder, ileum and lymph nodes of HEV-infected pigs in the current study. Four pigs showed an increasing trend in AST and ALT levels, which might suggest a slowly progressive development of liver damage during HEV-infection. Due to absence of control pigs in the current study these abnormalities cannot be attributed conclusively to the HEV-infection. However, mild hepatic lesions were previously associated with subclinical hepatitis E in naturally infected pigs [17,36,37] and experimentally infected pigs [21,22,25]. Therefore, a subclinical HEV-infection in domestic pigs may initiate energy-requiring recovery processes, possibly at the expense of production aspects such as growth rate, feed conversion or time to first estrus. Effects of HEV-infections on litter size and preterm abortion in pregnant gilts were absent [25], but effects on the above-mentioned production parameters have not been examined. This information is needed to evaluate from an economical perspective on whether intervention strategies for the reduction of the HEV-incidence among pigs can be beneficial to pig farmers.

## Conclusion

The course of HEV infection in contact-infected pigs was characterized by estimation of onset and the duration of faecal HEV RNA excretion and viremia, time until antibody development, the course of AST/ALT-levels and localization of HEV in tissues and organs. HEV RNA was detected in 4 of 7 contact-infected pigs and may be a source for HEV-transmission. Furthermore, 32 of 60 meat samples contained HEV RNA, suggesting the possibility of foodborne transmission to humans via pork products. Additional studies are required to assess whether urine and meat contain infectious HEV and to assess the currently unknown public health risk by contact with pigs and by consumption of pork.

## Methods

### Virus

Hepatitis E virus was acquired from a liver sample of a naturally infected Dutch fattening pig and handled as described previously [20]. The strain belonged to genotype 3.

### Experimental design

The infection experiment is described in detail elsewhere [20]. Briefly, this study was performed in two replicate blocks (blocks 1 and 2), each comprising 20 pigs of 3–4 weeks old at the start of the experiment. The HEV inoculum was prepared in bulk, aliquotted in 10 portions and stored at -70°C until use. The pigs were obtained from a conventional, SPF herd, free of the most significant pig pathogens (PRRSV, Actinobacillus pleuropneumonia, M. pneumoniae). Pigs were tested to be HEV RNA negative in faeces at two weeks and one week prior to inoculation. Furthermore, the pigs tested negative for anti-HEV antibodies in serum one week prior to the start of the experiment and on the day the experiment started. Blood samples were tested two weeks before start of the experiment to be negative for PCV2 and PRRSV by molecular methods.

Four pigs were allotted to each of five stables (~9 m^2 ^each) of a BSL2 facility, each stable containing three compartments. One pig was placed in each of two compartments and two pigs were placed in the third compartment. One of these two pigs was inoculated intravenously (*iv*-pig) with an estimated amount of ~10^4 ^PCR detectable units of HEV, while the other pig served as first-generation contact pig (C1-pig). Faecal samples were taken three times per week from the *iv*-pig and the C1-pig, and HEV excretion was monitored by conventional RT-PCR. When the C1-pig excreted HEV in faeces at three consecutive samplings it was moved to the compartment containing the second-generation contact pig (C2-pig). When the C2-pig excreted HEV in faeces at three consecutive samplings it was moved to the compartment containing the third-generation contact pig (C3-pig).

The experiment was approved by the institutes' animal ethical committee according to the Dutch law on animal experiments.

All 10 *iv-*pigs were HEV-infected and transmitted HEV to the C1-pigs by one-to-one exposure (Table 4). However, only eight of ten trials could be included in the analysis of HEV transmission from an infectious C1-pig to a C2-pig, because one infected C1-pig excreted HEV in faeces at three consecutive samplings, but then not from the day of transfer onwards, and faecal HEV RNA excretion was detected in one C2-pig prior to first exposure to an infected C1-pig (referred to as indirect contact infection). Seven of these C1-C2 trials were successful. However, because two C3-pigs were already non-contact infected and therefore out of the transmission analysis, and the indirectly infected C2-pig could be used for the analysis of HEV-transmission to a C3-pig, six trials were conducted to transmit HEV from a C2-pig to a C3-pig instead of seven. Of these six C2-C3 trials, five were successful. For two of the three indirect-contact infected pigs the onset of faecal HEV RNA excretion was known, and these pigs were therefore included in the current analyses. This yields a total of 24 pigs that were contact-infected (direct or indirect through an unknown route).

**Table 4:** Number of pigs that were HEV-infected due to infection routes other than *iv *in the experiment.

	Direct transmission			Indirect transmission
		
Type of pig	Transmission type	Exposed pigs	Infected pigs	
C1	*iv *→ C1	10	10	0
C2	C1 → C2	8	7	1
C3	C2 → C3	6	5	2*

No. of pigs used in the analyses			22	2

* for one of the two pigs, the moment of becoming infected was unknown and could therefore not be used in the analyses.

### Sampling

Faecal samples were taken and processed as described elsewhere [20]. Serum samples were collected every three to four days (twice per week) and centrifuged at 2,500 *g *for 10 min to obtain serum. Part of the serum was stored at -20°C for antibody detection at a later time, the remainder at -70°C for RNA extraction at a later time. Blood samples were collected only from those pigs that were housed together in one compartment (*i.e*. where HEV transmission was analyzed). In addition, when a contact-infected pig showed HEV RNA excretion at two consecutive samplings, a control sample was taken from the next-generation contact pig. Faeces and serum were collected from two *iv*-pigs up until 56 dpi in Block 1 and from two C1-pigs up until 55 and 65 days after first faecal HEV RNA excretion in Block 2.

Five *iv*-pigs were necropsied at 28 dpi, five C2-pigs at 25 or 26 days after first faecal HEV RNA excretion (referred to as 28 days postinfection), two *iv-*pigs at 56 dpi in block 1 and two C1-pigs at 55 and 65 days after first faecal HEV RNA excretion in block 2. The five *iv*-pigs and C2-pigs (three each in block 1 and two each in block 2) were appointed by randomly selecting one of the five stables. The two *iv*-pigs necropsied at 56 dpi were necessarily the two remaining *iv*-pigs in block 1. The two necropsied C1-pigs in block two came from the same stable as the two *iv*-pigs necropsied at 56 dpi in block 1. Pigs were sedated by injecting a high dose of barbiturates via the ear vein and were subsequently bled. During necropsy the following samples were collected in the specified order: blood, urine (directly from the bladder with a sterile syringe and needle), the longissimus muscle (pork chop), the iliopsoas muscle (tenderloin), the biceps femoris muscle (pork steak), tonsil, lymph nodes (bronchial, mesenterial and hepatic), pancreas, spleen, kidney, ileum, jejunum, colon, faeces, bile (directly from the gall-bladder with a sterile syringe and needle) and liver. Part of each tissue sample was fixed immediately in 10% neutral buffered formalin for histology and the other part was stored in tubes at -70°C upon return to the laboratory for RNA extraction at a later time.

### RNA extraction

RNA was extracted in a laboratory dedicated only to RNA extraction, from 140 μl of a 10% faecal suspension, serum or urine using the QiaAmp viral RNA minikit (Qiagen, Venlo, The Netherlands) according to the spin protocol supplied by the manufacturer. For bile samples, RNA was extracted from 100 μl using TRIzol (Invitrogen, Breda, The Netherlands). The RNA was eluted in a final volume of 35 μl elution buffer. For all tissue samples, RNA was extracted with an optimized protocol for liver samples using mechanical disruption of tissue samples with zirconium beads and subsequent silica-based RNA extraction, as described elsewhere [10].

### RT-PCR

The reagents for the RT-PCR were prepared in a laboratory dedicated to preparing reagents for (RT-)PCR, and the RNA was added in a separate laboratory dedicated to adding RNA to the (RT-)PCR reagents. The actual RT-PCR was performed in a third laboratory that contains only thermocyclers. This segregation of laboratories, combined with the experience of the lab technician, should reduce the likelihood of RT-PCR cross-contamination.

HEV RNA in all samples was detected using a real-time RT-PCR assay targeting ORF3 [38]. This protocol was modified to analyze 5 μl of RNA by increasing the amount of 2× QuantiTect Probe RT-PCR Master Mix (Qiagen) to 12.5 μl and the amount of enzyme to 0.24 μl per reaction. For confirmation, nine real-time PCRs that generated a threshold cycle (C_t_) > 38 were subjected to gel electrophoresis, showing fragments of the expected size. Non-template controls were included in each assay to monitor PCR contamination. During the experiment samples were analyzed using conventional RT-PCR directed at *open reading frame *2 (ORF2), as described previously [19]. Decisions for transfer of pigs to other compartments and the point of autopsy at 28 days post infection for C2-pigs were based on this conventional RT-PCR. After the experiment, 588 of the 591 faecal samples were re-examined for HEV RNA with the real-time ORF3 RT-PCR used for the other samples to enable direct comparison. Of the retested faecal samples, 278 samples tested negatively in both assays and 260 samples tested positively in both assays. Twelve of the conventional RT-PCR positive samples were negative by real-time RT-PCR; 38 of the real-time RT-PCR positive samples were negative by conventional RT-PCR. These additional positive samples were frequently observed just before or after a series of positive samples as determined by conventional RT-PCR.

Undiluted RNA samples were examined and if samples tested HEV-negative, then undiluted RNA and 10-fold diluted RNA samples were tested to dilute possible amplification inhibitors in the RT-PCR assay.

### Sequencing

RT-PCR fragments of the expected size were either excised from the agarose gel or were directly purified with a mini quick spin DNA column (Roche Diagnostics, Almere, The Netherlands), inserted in the pCRII-TOPO cloning vector (Invitrogen, Breda, The Netherlands), and transformed into chemically competent E. coli JM109 (Promega, Leiden, The Netherlands). After an incubation of 20 ± 4 h at 37 C, white colonies were examined for insertion of the correct RT-PCR fragment by PCR with M13 forward and reverse primers. PCR products were analyzed by electrophoresis, for the determination of the expected size, and were hybridized with a HEV-specific probe. Positive PCR products were purified with the QIAquick PCR purification kit (Qiagen, Venlo, The Netherlands). Sequencing was done with the BigDye Terminator Cycle Sequencing Ready Reaction (Perkin Elmer, Applied Biosystems, Foster City, Calif.).

### ELISA

Serum samples were examined for total anti-HEV antibodies (IgTot) with a double-antigen sandwich ELISA obtained from MP Biomedicals Asia Pacific Pte Ltd. in Singapore [39]. Samples with a sample to cut-off ratio ≥ 1 were considered positive. The cut-off value equaled 0.2 plus the mean optical density (OD) of negative controls.

### Clinical chemistry

Alanine aminotransferase (ALT) and aspartate aminotransferase (AST) levels were analyzed longitudinally by a spectrophotometric method in an automated analyzer (HumaStar 89, Instruchemie, Delfzijl, The Netherlands) for five C2-pigs and five *iv*-pigs necropsied at 28 days postinfection, and from the two C1-pigs necropsied at the end of Block 2. The reference values in normal feeder pigs used for ALT were 15 – 46 U l^-1 ^and for AST 16 – 67 U l^-1^.

### Histopathology

Samples of liver, gallbladder, ileum, spleen, mesenterial lymph nodes, kidney and pancreas collected from five C2-pigs and five *iv*-pigs at 28 days post infection, from the two *iv*-pigs necropsied at 56 dpi and the two C1-pigs necropsied at 55 and 65 days after first faecal HEV RNA excretion. The formalin-fixed samples were embedded in paraffin wax using routine procedures, sectioned at 4 μm and stained with hematoxylin and eosin.

### Statistics

In total, five time-related parameters were estimated with survival analysis [40]. These include: time until first faecal HEV RNA excretion, time until first HEV-detection in serum, time until antibody development, duration of faecal HEV RNA excretion and duration of viremia. The period until first HEV-excretion for contact-pigs was estimated from days post exposure (dpe). For C1-pigs, exposure was assumed to start at the midpoint of the interval between the last HEV-negative and first HEV-positive faecal sample of the respective *iv*-pig. For C2- and C3-pigs, exposure started on the day the C1- or C2-pig, respectively, was introduced. All other time-related parameters were estimated relative to the first day of faecal HEV RNA excretion, which was assumed to start at the midpoint of the interval between the last HEV-negative and the first HEV-positive faecal sample. This relative comparison was chosen because the moment of infection for contact-infected pigs is unknown, impeding the use of dpi. By using the onset of faecal HEV RNA excretion as a reference point the course of infection is normalized for all pigs. This normalization makes comparisons between pigs more appropriate.

To assess differences between contact-infected and inoculated pigs, data of the C2- and C3-pigs were pooled, as exposure to HEV was assumed to be similar. The C1-pigs, however, joint compartments with *iv*-pigs from inoculation onwards, displaying a different exposure-pattern than C2- and C3-pigs. Therefore, statistical differences between C1-pigs and C2/3-pigs were examined.

The differences in Kaplan-Meier (KM) survival curves for the five parameters were tested using the Log-rank statistic (*α *= 0.05) for the three groups: C1-pigs, C2/3-pigs and *iv*-pigs. To draw conclusions on differences between contact-infected and *iv*-pigs while accounting for the variation between the two blocks, joint *p*-values were calculated from the *p*-values per parameter per block using Fischer's method of computing the statistic $-2\sum_{i=1}^{2}\ln(p_{i})$[41]. This statistic is chi-square distributed with degrees of freedom twice the number of *p*-values added.

Trends for increasing AST- and ALT-levels in time were assessed per pig by linear regression, with AST- or ALT-levels as continuous response variable and sampling number as continuous explanatory variable. All statistical analyses were done using SAS (version 9.1; SAS Institute, Cary, NC, USA).

## Abbreviations

ALT: alanine aminotransferase; AST: aspartate aminotransferase; C#-pig: #-generation contact-infected pig; C_t_: threshold cycle; dpi: days post infection; dpe: days post exposure; *iv*-pig: intravenously inoculated pig.

## Authors' contributions

MB conceived of the study, carried out the molecular and statistical analyses and drafted the manuscript. SR and ARH contributed to the molecular analysis and helped to finalize the manuscript. CR was responsible for the ELISAs. NS was responsible for the histopathology and enzyme analyses. MB, KF, MdJ, ARH and WvdP participated in the design of the study. WvdP coordinated the animal experiment. KF and MdJ participated in the statistical analyses. All authors contributed to interpretation of results, commented on earlier versions of the manuscript, and read and approved the final version.
